# The Gene *vitellogenin* Has Multiple Coordinating Effects on Social Organization 

**DOI:** 10.1371/journal.pbio.0050062

**Published:** 2007-03-06

**Authors:** C. Mindy Nelson, Kate E Ihle, M. Kim Fondrk, Robert E Page, Gro V Amdam

**Affiliations:** 1 Department of Entomology, University of California Davis, Davis, California, United States of America; 2 School of Life Sciences, Arizona State University, Tempe, Arizona, United States of America; University of London, United Kingdom; 3 Department of Animal and Aquacultural Sciences, Norwegian University of Life Sciences, Aas, Norway

## Abstract

Temporal division of labor and foraging specialization are key characteristics of honeybee social organization. Worker honeybees *(Apis mellifera)* initiate foraging for food around their third week of life and often specialize in collecting pollen or nectar before they die. Variation in these fundamental social traits correlates with variation in worker reproductive physiology. However, the genetic and hormonal mechanisms that mediate the control of social organization are not understood and remain a central question in social insect biology. Here we demonstrate that a yolk precursor gene, *vitellogenin,* affects a complex suite of social traits. Vitellogenin is a major reproductive protein in insects in general and a proposed endocrine factor in honeybees. We show by use of RNA interference (RNAi) that *vitellogenin* gene activity paces onset of foraging behavior, primes bees for specialized foraging tasks, and influences worker longevity. These findings support the view that the worker specializations that characterize hymenopteran sociality evolved through co-option of reproductive regulatory pathways. Further, they demonstrate for the first time how coordinated control of multiple social life-history traits can originate via the pleiotropic effects of a single gene that affects multiple physiological processes.

## Author Summary

Animals that live in groups often specialize in different tasks, creating a division of labor. One extreme example can be seen in honeybees, in which most tasks are performed by thousands of worker females that are essentially sterile helpers. Workers start out as nurse bees that care for larvae in the nest. Later they embark on foraging trips, specializing in either pollen or nectar collection, and continue to forage until they die. The age when workers initiate foraging and the tendency to collect pollen or nectar have been linked to a rudimentary reproductive physiology in which the protein vitellogenin appears to play a central role. Vitellogenin is normally used to produce egg yolk, but it may affect behavior and lifespan in workers. We tested this hypothesis by knocking down the *vitellogenin* gene of worker bees. Workers with suppressed vitellogenin levels foraged earlier, preferred nectar, and lived shorter lives. Thus, vitellogenin has multiple effects on honeybee social organization. By using gene knockdown to understand insect social behavior, our study supports the view that social life in bees evolved by co-opting genes involved in reproduction.

## Abstract 3

*vitellogenin* gene activity paces onset of foraging behavior in worker bees, demonstrating how coordinated control of multiple social life-history traits can originate via the pleiotropic effects of a single gene.

## Introduction

Vitellogenin has versatile regulatory functions in honeybees, suggesting that this glycolipoprotein may be involved in the control of social life-history traits [1–5]. Vitellogenin is the common yolk precursor protein of oviparous taxa [6]. However, it appears to have evolved pleiotropic functions in the advanced eusocial honeybee that have not as yet been given attention in other species that rely on vitellogenin for oocyte development [1,4].

Honeybee vitellogenin has been hypothesized to work together with juvenile hormone in a double repressor network to coordinate behavior [7]. In this network, vitellogenine suppresses juvenile hormone and inhibits the worker honeybees' age-associated shift from nest tasks to foraging duties [3]. This shift is a complex behavioral transition characterized by decreasing vitellogenin and increasing juvenile hormone titer [8]. It has been proposed also that variation in *vitellogenin* gene expression early in life is associated with subsequent behavioral specialization that gives rise to a division of labor between nectar and pollen foraging workers [9]. Finally, honeybee vitellogenin can reduce oxidative stress by scavenging free radicals, thereby prolonging lifespan in the facultatively sterile worker castes and the reproductive queen castes [4]. Similar antioxidant function has been suggested for vitellogenin molecules in the nematode Caenorhabditis elegans [10] and in eggs of the eel Anguilla japonica [11], but positive effects of vitellogenin on adult longevity have not been demonstrated in these species.

In summary, the proposed pleiotropic effects of honeybee vitellogenin suggest that the *vitellogenin* gene is a central element in the life-history regulation of this social insect. Here we test these proposed functions by using RNA interference (RNAi) to knock down expression of the honeybee *vitellogenin* gene.

## Results

**Figure 1:**
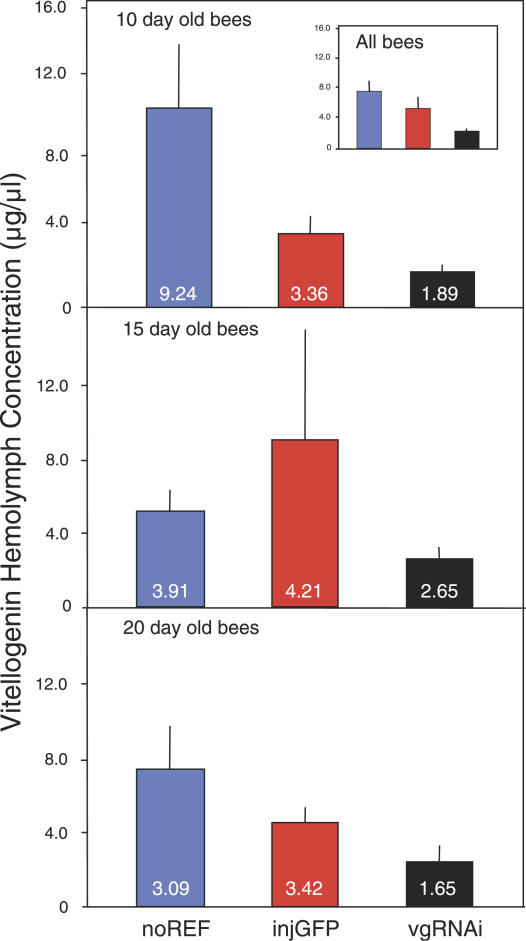
The Effect of *vitellogenin* RNAi on Hemolymph Vitellogenin Concentrations Levels are in micrograms per microliter relative to a β-galactosidase standard. Significant suppression of vitellogenin is apparent in RNAi knockdowns (vgRNAi) compared to injected controls (injGFP; Mann-Whitney *U* test: *Z* = 2.84, *n* = 54, *p* < 0.005). Control injections (GFP-derived dsRNA) did not significantly affect the vitellogenin level of the bees compared to the non-injected reference group (noREF; Mann-Whitney *U* test: *Z* = −1.10, *n* = 55, *p* = 0.27). Bars show results as means and standard errors with corresponding medians at the bottom of each bar. Because the dataset did not conform to assumptions of parametric tests (see Materials and Methods), medians can be considered the more accurate statistic. The dataset is split by age (10, 15 and 20 d olds) to visualize the persistence of RNAi. However, age did not affect the vitellogenin level of the workers (*p* = 0.68, see data analysis section for details), and thus conclusions cannot be drawn about treatment effects by age. The means and standard errors of the dataset overall are shown in the embedded box of the upper panel (medians for the dataset: noREF = 3.94, injGFP = 3.45, and vgRNAi = 2.46).

The *vitellogenin* RNAi tool [12] was used in combination with observations of the behavior and lifespan of worker honeybees living in otherwise unmanipulated colonies. RNAi-mediated knockdown of the vitellogenin protein has been confirmed repeatedly in 5–7-d-old worker bees [3,5,12]. In this first RNAi study of honeybee social life history, however, we aimed to monitor workers over several weeks. Therefore, RNAi was validated in cohorts of 10-d-old (*n* = 31), 15-d-old (*n* = 27) and 20-d-old (*n* = 27) bees (Figure 1).

This initial test demonstrated that workers with a *vitellogenin* RNAi phenotype (*n* = 30) were characterized by persistent suppression of vitellogenin protein levels compared to controls (*n* = 24), which received injections of double-stranded RNA (dsRNA) derived from green fluorescent protein (GFP) encoding sequence (*p* < 0.005; see the legend to Figure 1 for details on statistics). The GFP dsRNA control represents a handling disturbance control [5], which is necessary because honeybees respond to many kinds of handling stress with changes in endocrines, neuromodulators, and behavior [13–15]. This control was monitored relative to a non-injected reference group (*n* = 31; see Figure 1 for details). The vitellogenin levels of the GFP dsRNA control and the non-injected reference group were not significantly different (*p* = 0.27).

**Figure 2:**
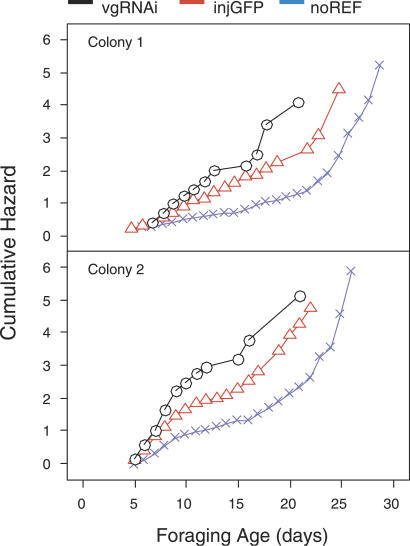
Effect of *vitellogenin* Gene Activity on Age at Foraging Onset Cumulative hazard increases until all bees have foraged. Knockdowns (vgRNAi) initiated foraging behavior earlier in life than injected controls (injGFP; LRT = 8.81, *df* = 1, *p* < 0.003, hazard ratio [comparative risk of initiating foraging calculated over the entire time of the study] = 1.43 d earlier, confidence interval = 1.13–1.81 d). A documented effect of laboratory handling [13,14] is shown by the injected controls foraging earlier than the non-injected reference (noREF) workers (LRT = 44.0, *df* = 1, *p* < 0.0001, hazard ratio = 1.97 d earlier, confidence interval = 1.62–2.39 d).

Next, we found that *vitellogenin* knockdowns (*n* = 122) initiated foraging flights earlier in life than GFP dsRNA controls (*n* = 179, *p* < 0.003; see Figure 2 for details). These results confirm the hypothesis that honeybee *vitellogenin* gene activity influences worker division of labor via an inhibitory effect on the shift from nest tasks to foraging [3,7].

**Figure 3:**
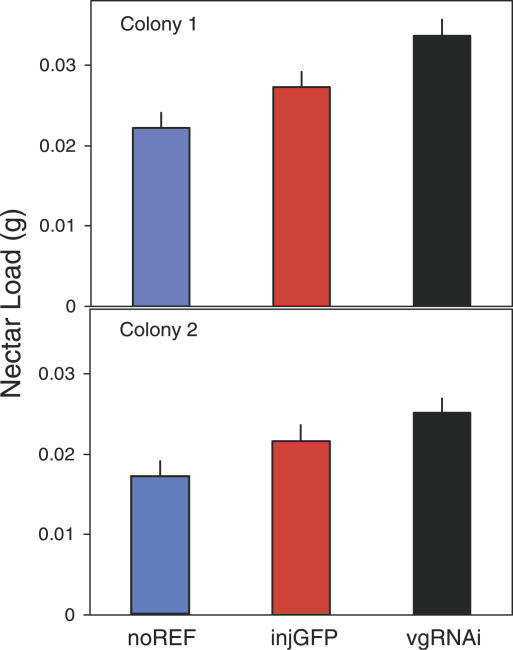
Effect of *vitellogenin* Gene Activity on Size of Nectar Loads Collected Knockdowns (vgRNAi) collected more nectar than injected controls (injGFP; ANOVA, *F*_1,315_ = 6.79, *p* < 0.010). The difference between injected controls and non-injected reference (noREF) bees was also significant (ANOVA, *F*_1,313_ = 6.00, *p*< 0.015). Bars are means with standard errors.

In addition, down-regulation of *vitellogenin* gene activity (*n* = 160) resulted in foragers collecting larger loads of nectar relative to GFP dsRNA controls (*n* = 159, *p* < 0.010; see Figure 3 for details). Overall, loads were within the range normally collected by honeybees (up to 60-mg nectar and 30-mg pollen [16,17]), and thus interfering with *vitellogenin* expression did not change the maximum load size collected by workers. The observed bias towards nectar collection in *vitellogenin* knockdowns is consistent with earlier studies showing low hemolymph (blood) levels of vitellogenin in young worker bees from genetic stocks that preferentially collect nectar [9]. Genetic stocks with bias for collecting pollen are characterized by high levels of vitellogenin prior to foraging onset [9]. Our data, however, go beyond these correlations and demonstrate that the *vitellogenin* gene influences social foraging specialization.

**Figure 4:**
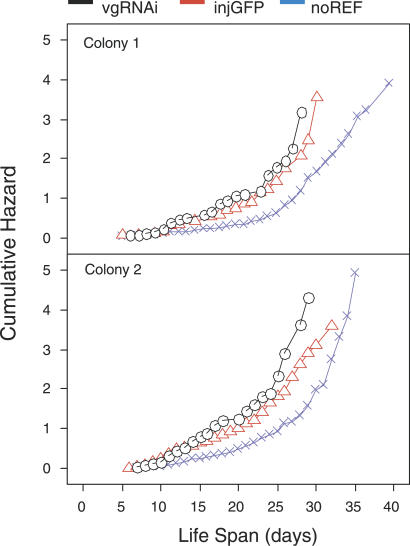
Effect of *vitellogenin* Gene Activity on Lifespan Cumulative hazard increases until all bees have died. Injected controls (injGFP) lived longer than knockdowns (vgRNAi; LRT = 4.38, *df* = 1, *p* < 0.036, hazard ratio [comparative survival experience calculated over the entire time of the study] = 1.29 d longer, confidence interval = 1.02–1.63 d). Longevity was affected by laboratory handling (LRT = 43.4, *df* = 1, *p* < 0.0001, hazard ratio = 1.96 d longer for non-injected reference (noREF) bees compared with injGFP bees, confidence interval = 1.61–2.38 d).

Survival data showed that vitellogenin also is involved in the regulation of honeybee lifespan. Lifespan was reduced in *vitellogenin* knockdowns (*n* = 122) compared with GFP dsRNA controls (*n* = 179, *p* < 0.036, see Figure 4 for details). The effect was not due simply to bees initiating foraging behavior earlier in life, because these traits were not correlated in the knockdown phenotype (*r* = 0.121 [Colony 1]; *r* = −0.003 [Colony 2], *p* > 0.05). Our finding is supported by the previous results showing that worker bees with reduced *vitellogenin* activity levels are more susceptible to oxidative stress [4], a physiological state that is an established indicator of aging [18,19].

## Discussion

**Figure 5:**
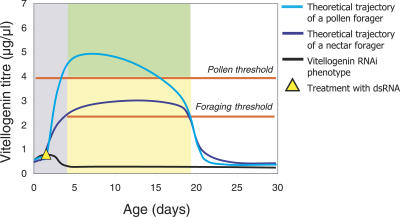
Vitellogenin Has a Dual Role in Regulation of Social Behavior After the maturation phase, when bees are unable to forage (grey) [37], vitellogenin suppresses the transition from nest tasks to foraging activity when its titer remains above the foraging threshold level [3,7]. Below this threshold, the probability of initiating foraging is increased [5]. Pre-foraging vitellogenin titers above the pollen threshold prime workers for pollen foraging (green), while workers with lower pre-foraging titers (yellow) are primed for nectar foraging [9]. *vitellogenin* RNAi causes workers to mature with vitellogenin titers that are below both thresholds [3,7,9,12], resulting in the *vitellogenin* knockdown phenotype documented here: bees that forage precociously and preferentially collect nectar.

Our results suggest that honeybee vitellogenin has an integrative function in regulating social organization through its pleiotropic effects on division of labor and foraging specialization. Vitellogenin inhibits the onset of foraging (our study) but declines with age in workers [8,20], thereby serving as a pacemaker for age polyethism and lifespan, as first hypothesized by Omholt and Amdam [7,21]. Higher titers early in life [9] prime bees for pollen collection, whereas low titers prime bees for collecting nectar (our study). *vitellogenin* RNAi established at adult emergence triggers persistent suppression of *vitellogenin* activity [3,5,12] (Figure 1), and therefore, the knockdown phenotype was expected to initiate foraging early, collect nectar, and live a short life (Figure 5).

Life-history pleiotropy demonstrated by the effects of vitellogenin is similar in principle to trait associations that are controlled by the systemic endocrine factors juvenile hormone and ecdysone in the solitary insect *Drosophila* (reviewed by Flatt et al. [22]). In *Drosophila,* yolk precursor peptides [23] are downstream components of these hormonal signaling cascades [24], whereas in honeybees, *vitellogenin* is part of a regulatory feedback loop that enables vitellogenin and juvenile hormone to mutually suppress each other [3,4,7]. As a consequence, vitellogenin and juvenile hormone should be considered joint effectors of division of labor and foraging specialization, at least until methods can be developed to separate their individual effects. This feedback relationship is uncommon in insects [3], suggesting in combination with our findings that evolutionary co-option and remodeling of vitellogenin and juvenile hormone action [1,4–6] have been important steps in honeybee social evolution [3].

Previous studies have identified genes that affect honeybee foraging onset (*Amfor* [25] and *malvolio* [26]), and multiple genes with mRNA levels that correlate with foraging behavior [27] or lifespan [28]. Yet our work represents the first successful RNAi approach to decipher gene and protein function in honeybee social behavior. Our data demonstrate for the first time that several key characteristics of a social phenotype can be coordinated by a single reproductive gene. This pleiotropy lends support to studies showing that complex social behavior in insects can evolve from ancestral reproductive traits [9,29–31].

## Materials and Methods

### Validation of vitellogenin knockdown.

The non-injected reference (noREF), GFP dsRNA control (injGFP), and *vitellogenin* knockdown (vgRNAi) phenotypes were obtained as previously described by Amdam et al. [5,12]. In short, primers were designed from the sequence of the *A. mellifera vitellogenin* cDNA clone *AP4a5,* and the GFP encoding sequence of the pGFP vector (Clontech, Palo Alto, California, United States). GFP dsRNA does not affect vitellogenin [5], but was used in planned comparisons with *vitellogenin* dsRNA to control for laboratory handling that affects sensory and physiological correlates of worker foraging behavior [5,13,14].

Primers were fused with T7 promoter sequence (underlined): for clone *AP4a5*: 5′-TAATACGACTCACTATAGGGCGAACGACTCGACCAACGACTT-3′ and 5′-TAATACGACTCACTATAGGGCGAAACGAAAGGAACGGTCAATTCC-3′; and for pGFP: 5′-TAATACGACTCACTATAGGGCGATTCCATGGCCAACACTTGTCC-3′ and 5′-TAATACGACTCACTATAGGGCGATCAAGAAGGACCATGTGGTC-3′. PCR reactions were performed according to standard procedures using *AP4a5* and the pGFP vector as templates. Resulting products excluding the fused T7 promoters were 504 base pairs (bp) (*vitellogenin*) and 503 bp (GFP derived). Products were purified using the QIAquick PCR purification kit (Qiagen, Valencia, California, United States), and RNA was prepared using the Promega RiboMax T7 system (Promega, Madison, Wisconsin, United States). RNA was extracted by TRIzol LS reagent (GIBCO-BRL, San Diego, California, United States), resuspended in nuclease-free water, heated at 96 °C for 2 min in an Eppendorf Thermomixer (Brinkmann Instruments, Westbury, New York, United States), and left to cool at room temperature for 20 min. The integrity of the dsRNA was tested using 1.5% agarose gels, and the products were diluted with nuclease-free water (Qiagen) to the final concentration of 5 μg/μl.

Newly emerged workers were randomly assigned to one of three treatments and marked with paint (Testors Enamel; Testor Corporation, Rockford, Illinois, United States) to indicate treatment identity. The noREF group was set aside. The two remaining groups were injected with *vitellogenin*-derived or GFP-derived dsRNA (to make up the vgRNAi or injGFP treatment, respectively) between the fifth and sixth tergite using Hamilton syringes with G30 disposable needles (BD, Palo Alto, California, United States). Injection volume was 2 μl. Injections were performed by trained personnel that were blind to treatment identities. Workers were introduced into two colonies kept in commercial hive boxes (*n* = 50 for each treatment group and in each colony, respectively) and collected after 10, 15, and 20 d during non-foraging hours.

Bees were anesthetized on ice, and hemolymph was extracted with Drummond Scientific Company (Broomall, Pennsylvania, United States) micropipettes after puncturing the abdomen between the third and fourth tergite with a sterile G30 needle (BD). Care was taken to avoid contaminating the samples with tissue fragments and foregut content. Hemolymph (1 μl) was dissolved in 10-μl Tris buffer: 20 mM Tris, 150 mM NaCl, 5 mM EDTA (pH 7.5), 1 mM phenylmethylsulfonyl fluoride, 5 mM benzamidin, 0.7 μM pepstatin, 8 μM chymostatin, 10 μM leupeptin, 0.8 μM aprotinin (Sigma-Aldrich, St. Louis, Missouri, United States), before samples were separated by 8% SDS-polyacrylamide gel electrophoresis using standard methods [32]. A β-galactosidase standard (Sigma-Aldrich) was included to allow densitometrically quantification by the method of Lin et al. [33], in which vitellogenin is detected as a single band of 180 kDa [5,33,34]. The densitometrical analysis was performed by the Quantity One imaging software (Bio-Rad, Hercules, California, United States) after staining the gels with Commassie Brilliant Blue (Sigma-Aldrich). Gel-to-gel variation in staining intensity was controlled by background correction and the β-galactosidase standards as we have described previously [5].

### Age of foraging onset and lifespan.

Newly emerged workers were randomly assigned to one of three treatments. noREF bees were uniquely tagged and set aside. Groups of vgRNAi and injGFP workers were obtained as described above. About equal numbers of bees from each treatment group were introduced into two host colonies *(n* = 288 noREF, 338 injGFP, and 293 vgRNAi into Colony 1; and *n* = 288 noREF, 338 injGFP, and 299 vgRNAi into Colony 2). Each colony was set up in an observation hive with about 5,000 unmarked adult bees of diverse ages. Observers were blind to treatment identity. Colonies were surveyed daily for tagged bees during non-foraging hours to establish survivorship (the combs of each colony were scanned twice daily), and for two 40-min periods daily during peak foraging hours to establish foraging activity. Ramps from the outside port leading to the bottom comb were observed for incoming tagged forager bees during these 40-min sessions. A bee's age of foraging onset was the number of days since adult emergence that she was first seen returning from a foraging trip. A bee's day of death was established as the day after the last day she was observed.

### Substance collected.

Set-ups were separate, but identical, to the one described for age of foraging onset and lifespan, except that host colonies were kept in commercial hive boxes. During peak foraging hours, incoming foragers were collected at the hive entrances, brought to the laboratory, and sampled once for type of substance collected. Bees were not returned to the colonies after sampling. At the time of collection, the workers were 10–16 d old (Colony 1) or 7–13 d old (Colony 2). Pollen and/or nectar loads were removed and quantified for each individual worker as described before [35]. In all, *n* = 80 noREF, 84 injGFP, and 65 vgRNAi bees were taken from Colony 1, and *n* = 78 noREF, 75 injGFP, and 95 vgRNAi bees were taken from Colony 2. Workers that returned empty (with no measurable nectar or pollen) were not used in our analyses.

### Data analysis.

The dataset on hemolymph vitellogenin levels was not normally distributed, as determined by Bartlett's test of sphericity. Therefore, the non-parametric Kruskal-Wallis test was used (*n* = 85) to examine the data for effects of treatment (*H* = 14.32, *p* = 0.001) and age (*H* = 0.79, *p* = 0.68). A Mann-Whitney *U* test was used to test for colony effects (*Z* = −3.54, *p* < 0.001). Vitellogenin titers were higher overall in Colony 2, but the relative expression pattern between treatments was the same in the two colonies (unpublished data). Therefore, the dataset was not split by colony, but used in full to increase the statistical power of the post hoc analysis. A Mann-Whitney *U* test was used as a post hoc test of the planned comparisons of vitellogenin levels between noREF and injGFP, and between injGFP and vgRNAi. Treatment differences in age of foraging onset and lifespan were analyzed using the Cox proportional hazards regression model with the likelihood ratio chi-square tests (LRT) for bees that foraged at least twice (*n* = 149 noREF, 69 injGFP, and 48 vgRNAi for Colony 1; and *n* = 137 noREF, 110 injGFP, and 74 vgRNAi for Colony 2). The proportional hazards assumption was verified by Schoenfeld residuals [36]. Colony effects were controlled for by using the colony as a stratifying variable in the analyses. Effect of treatment was detected in the dataset overall (foraging onset LRT = 95.1, *df* = 2, *p* < 0.0001; lifespan LRT = 82.2, *df* = 2, *p* < 0.0001). Thus, planned pair-wise comparisons were made between noREF and injGFP, and between injGFP and vgRNAi. The data on foraging loads passed Bartlett's test of sphericity. Two-way analysis of variance (ANOVA) was therefore used to test for effects of treatment on foraging loads while controlling for the colony factor. A significant effect of treatment was detected (ANOVA, *F*_2,471_ *p* < 0.0001) and planned pair-wise comparisons were made using a two-way ANOVA. Pearson analysis was used to correlate age at foraging onset and lifespan. Statistical software was SPLUS version 6.1 (http://www.insightful.com), Systat 6.0 (http://www.systat.com), and Statistica 6.0 (http://www.statsoft.com).

## Supporting Information

### Accession Numbers

The GenBank (http://www.ncbi.nlm.nih.gov/Genbank) accession numbers for the genes discussed in this paper are *vitellogenin* (AJ517411) and the GFP encoding sequence of the pGFP vector (AF324407).

## Abbreviations

ANOVA: analysis of variance
dsRNA: double-stranded RNA
GFP: green fluorescent protein
injGFP: control bees injected with GFP-derived dsRNA
LRT: likelihood ratio chi-square test
noREF: non-injected reference bees
RNAi: RNA interference
vgRNAi: *vitellogenin* knockdown bees
